# Subjective Understanding is Reduced by Mechanistic Framing

**DOI:** 10.5334/joc.393

**Published:** 2024-07-24

**Authors:** Jeffrey C. Zemla, Daniel Corral

**Affiliations:** 1 Department of Psychology, Syracuse University, US

**Keywords:** understanding, metacognition, reasoning, explanation

## Abstract

People often believe that they have a good understanding of how devices work (e.g., how a ballpoint pen works), despite having poor knowledge of their internal mechanics. We hypothesized that this bias occurs in part because people conflate mechanistic understanding with functional understanding of how devices work (e.g., how to operate a ballpoint pen). In two experiments, we found that increasing the salience of mechanistic information led to lower judgments of understanding for how devices work. In Experiment 1, we did this by showing participants either the internal parts of a device or an external, whole-object view of that same device. Those who saw the internal parts rated their understanding as less than those who saw a whole-object view. In Experiment 2, we removed the pictures and instead tested participants (without feedback) on their mechanistic or functional knowledge using true-or-false questions. Those who were tested on mechanistic knowledge rated their understanding of devices as less than those who were tested on functional knowledge.

The drive to understand how things work is a core feature of human cognition (Gopnik, 2000). Nevertheless, the ability to assess one’s understanding of complex systems is not well calibrated with one’s actual understanding. People routinely overestimate their own understanding (Rozenblit & Keil, 2002) and fail to consider what they do not know when making metacognitive judgments (Walters et al., 2017). Acquiring a deep understanding of a phenomenon is cognitively demanding and time-intensive, yet people commonly assess their own understanding in seconds using fast and associative mnemonic cues, such as familiarity and ease of processing (Koriat, 1997).

## Subjective Understanding is Not Well Calibrated

Superficial and irrelevant factors can affect one’s understanding. Studies have found that *seductive details*–details that are interesting but irrelevant to the learning goal–can negatively impact comprehension (Garner et al., 1989; Harp & Mayer, 1997). For example, Harp and Maslich (2005) gave participants a lecture about how lightning forms that either was or was not interspersed with additional seductive details (e.g., “Every year lightning kills approximately 150 Americans”). Those who listened to the lecture with seductive details recalled fewer details about how lightning forms (e.g., “Raindrops and ice crystals drag air from in the cloud downward”) than those who did not. The influence of seductive details extends to metacognitive judgments of understanding as well. For example, Ikeda et al. (2013) found that people rated their understanding of neuroscientific phenomena as higher when images of brain activation were included relative to those who saw a bar graph containing the same information. Similarly, people prefer explanations of psychological phenomena that include irrelevant neuroscientific details to those that do not (Weisberg et al., 2008). In another study, participants who watched a trivially informative video of a pilot landing a plane rated their confidence in being able to land a plane safely as higher than participants who did not watch this video, though a professional pilot called the video “100% useless” (Jordan et al., 2022).

Other research has shown that metacognitive judgments of understanding are biased by what is knowable, as opposed to what one actually knows. For example, people conflate their own understanding of phenomena with other people’s, believing that they understand something better when told scientists understand it, compared to when they do not (Sloman & Rabb, 2016). Similarly, people believe that they understand things better when they have access to information (e.g., on the Internet) compared to when they do not (Fisher et al., 2015; Fisher & Oppenheimer, 2021).

## Familiarity and Ease of Processing Influence Subjective Understanding

A complete explanation for why people overestimate their understanding is multifaceted, but one reason is that memory for highly familiar objects is often incomplete and lacks details. Nickerson and Adams (1979) found that people were remarkably bad at drawing a U.S. penny from memory, omitting key details and getting relational aspects incorrect (such as whether the text “*one cent*” is above or below Lincoln). When shown fifteen drawings of pennies (one real, fourteen fake), fewer than half of participants correctly recognized the true penny. In related work, Lawson (2006) found that most people, including many experts, could not accurately draw or identify the position of the pedals, chain, and frame on a bicycle. Critically, familiarity with common objects can induce high processing fluency (Alter & Oppenheimer, 2009), generating a belief that we understand such objects better than we actually do (Kelley, & Lindsay, 1993).

Though people do not typically have a complete understanding of how objects work, they may still possess a partial understanding (Haskel-Ittah, 2023; Keil, 2006, 2019). That is, people may possess an understanding that contains gaps, or an understanding at a high level but not when decomposed into constituent parts. Often, a partial understanding of how something works is sufficient for people to navigate the world. A complete understanding of how a bicycle works is not necessary to ride a bicycle, to diagnose why a bicycle will not function (e.g., a loose chain), or to search for information on how to fix a bicycle.

## Conflating Functional Understanding with Mechanistic Understanding

Partial understanding may allow us to confuse a high-level, functional understanding of how to operate a device with a low-level, mechanistic understanding of how the parts of a device interact to produce a behavior. In this sense, overconfidence in understanding may be a form of attribute substitution (Kahneman & Frederick, 2002), where people substitute a complex question with one that is simpler and easier to answer. The English language conflates these multiple senses of understanding. For example, someone trying to pair their mobile phone to a Bluetooth speaker may ask, “*How does this work?*” Pragmatism suggests that in such cases, people are not asking for an explanation involving short-range radio waves, but are instead asking for instructions on how to operate the device.

Mechanistic and functional thinking are believed to represent different construals, or mental modes of understanding (Keil, 2006; Lombrozo, & Wilkenfeld, 2019). Preference for functional or mechanistic explanations depends in part on which construal the context affords (Vasilyeva, Wilkenfeld, & Lombrozo, 2017). However, a predisposition towards functional explanations emerges early in childhood and continues in adulthood for some domains, including artifacts (Kelemen, 1999a). A partial understanding of mechanism combined with a predisposition to explain function may lead people to adopt a functional construal to ambiguous phrasings such as “How does this work?”

Previous research supports the hypothesis that overconfidence is driven in part by a confusion of functional and mechanistic understanding. Rozenblit and Keil (2002) found that many people believe that they understand how mechanical devices work (e.g., how a ballpoint pen works), but when prompted to explain how the device in question works, they often fail to do so. After explaining, people who re-rate their subjective understanding typically provide lower ratings, a phenomenon known as the *illusion of explanatory depth*. The authors primarily used man-made artifacts as stimuli (e.g., a sewing machine, a toilet), but report one study in which they also used natural phenomena (e.g., earthquakes, tides). Since only artifacts have prescribed functions, they reasoned that the illusion might not persist for natural phenomena because conflation of functional and mechanistic understanding is not possible. While Rozenblit and Keil (2002) found that an illusion of explanatory depth *does* exist for naturally occurring phenomena, a marginally significant interaction for this effect and domain^1^ (*p* < .07) was also observed, suggesting that overconfidence is larger in devices than natural phenomena. The authors conclude that this result “suggest[s] a function mechanism confusion as a small factor in overconfidence for devices” (Rozenblit & Keil, 2002, p. 546; see also Keil, 2019).

Other work on the illusion of explanatory depth provides supporting evidence for this hypothesis. Alter et al. (2010) found that people are better calibrated, resulting in a smaller illusion of explanatory depth, when asked to rate their understanding of *how the parts of a ballpoint pen enable it to work* as opposed to *how a ballpoint pen works*. This finding demonstrates that when judgments of understanding are phrased explicitly to avoid ambiguity (emphasizing *parts* implies mechanistic knowledge; McCarthy & Keil, 2023), participants report lower levels of understanding than when the question is ambiguous and are more consistent in their ratings across two timepoints. In another study, participants were asked to report their understanding of political policies (Fernbach, Rogers, et al., 2013). Afterwards, participants either listed reasons for that policy (appealing to function) or explained how the policy works (appealing to mechanism) and then re-rated their understanding. The results showed that explaining the mechanisms of a policy caused a significant reduction in understanding ratings, whereas listing reasons did not. One possible explanation for this result is that participants interpret their initial judgment of understanding as one of functional understanding, and re-evaluate this interpretation only when primed to think about the corresponding mechanisms.

Collectively, this work leads to the prediction that there is a negative relationship between the saliency of mechanistic information and judgments of understanding. Specifically, judgments of understanding for artifacts should be lower when participants are primed to think about mechanistic information compared to when participants are not primed to think about mechanisms. However, some of this same work also provides evidence that casts doubt on this hypothesis.

For example, Rozenblit and Keil (2002) find a strong positive correlation between initial judgments of understanding and the ratio of visible to hidden parts of a device. The functional-mechanistic confusion hypothesis predicts the opposite: since a device with many visible parts (e.g., a bicycle) directly exposes its mechanisms, visibility of those parts should lead to *lower* judgments of understanding. One confound, however, is that devices with a high ratio of visible to hidden parts tend to have fewer parts overall (as reported by Rozenblit & Keil, 2002), whereas devices with more parts overall (e.g., a television) tend to hide them. Critically, objects that fit into this latter category may objectively be more difficult to understand because they consist of more parts. In our Experiment 1, we re-visit this possibility by asking two groups of participants to rate their understanding of the *same devices*, varying only whether an image of the device shows its internal parts or not. With this design, the objective complexity of the device is kept constant.

In another experiment reported by Alter et al. (2010), the authors induce either an abstract or a concrete mindset by asking participants to explain *why* or *how* they perform certain actions (e.g., drive a car, get dressed, and backup a computer) prior to completing an illusion of explanatory depth experiment. Though intended for different purposes, this manipulation should also induce either a functional or mechanistic mindset and we would expect it to influence initial understanding judgments of devices. While this manipulation significantly affected the magnitude of the illusion of explanatory depth, it had no impact on initial judgments of understanding.

One possible reason for this outcome is that the *how* condition essentially asked participants to write a behavioral procedure and not describe internal mechanisms. For example, describing how to drive a car is very different than describing how the internal mechanisms of a car work. Rozenblit and Keil (2002) note that subjective understanding of procedures is less susceptible to miscalibration because they are routine and more accessible in memory. Another possibility is that participants in Alter et al. (2010), as in Rozenblit and Keil (2002), were given a clear and detailed example of a mechanistic explanation for how a ballpoint pen works which provided participants an anchor for how this level of understanding relates to the Likert scale used to rate subjective understanding. As a result, ambiguity about whether functional or mechanistic understanding is being probed should be resolved prior to the initial understanding judgment.

While these studies suggest that prompts of “how” something works can lead to functional-mechanistic confusion, research on teleological reasoning and prompts of “why” something occurs may similarly lead to confusion. Kelemen (1999b) found that children prefer functional explanations (specifically, teleological ones) over mechanistic explanations to “why” questions pertaining to natural kinds, despite that natural kinds have no prescribed function or purpose. For example, first- and second-grade students tended to endorse explanations like “Rocks are pointy so that animals wouldn’t sit on them”. Though less pronounced, this bias towards teleological explanations extends to adults who are under time pressure and those who are less reflective (Kelemen & Rosset, 2009; Kelemen, Rottman, & Seston, 2013; Zemla, Steiner, & Sloman, 2016). These results suggest that functional explanations may be more accessible even when those explanations are scientifically unwarranted.

Joo, Yousif, and Keil (2022) directly addressed the ambiguity of “why” questions such as “Why does the mononykus [dinosaur] have such a long tail?” by asking participants what the questioner “really wanted to know.” Participants either rephrased the question or chose from alternate wordings of the question. For questions pertaining to artifacts, participants preferred phrasings that appealed to function, as one might expect. However, for non-living natural kinds, participants preferred non-functional phrasings, and for animals the results were mixed. The authors conclude that past claims about participants holding scientifically unwarranted beliefs may be overstated, and that participants may have been providing valid responses to a different interpretation of the questions themselves.

Much of the evidence we have presented both for and against the hypothesis of functional-mechanistic confusion comes from work on the illusion of explanatory depth. In these experiments, however, the authors are primarily not interested in testing the functional-mechanistic hypothesis, instead focusing on how self-explanation affects subjective understanding relative to pre-explanatory ratings. In fact, the classic illusion of explanatory depth paradigm presents participants with a detailed mechanistic explanation of how a ballpoint pen works prior to completing the main task (Rozenblit & Keil, 2002; Alter, Oppenheimer, & Zemla, 2010). As a result, any functional-mechanism confusion in this paradigm reflects a metacognitive error (considering the *wrong* mode of understanding). But in other contexts, it is more ambiguous whether functional or mechanistic understanding is being probed (Joo, Yousif, & Keil, 2022). In moving away from the illusion of explanatory depth paradigm, we explore more generally how people conceptualize their sense of understanding about artifacts and how a functional and mechanistic mindset affects that sense when providing metacognitive judgments.

We report two experiments testing the hypothesis that priming mechanistic knowledge affects judgments of understanding for man-made devices compared to a non-mechanistic framing. In Experiment 1, we use pictures of a device’s parts (compared to a whole-object view of the same device) to prime mechanistic thinking. We reasoned this procedure would encourage participants to think about mechanism because a mechanistic understanding of how a device works incorporates an understanding of the device’s parts and how they interact. In Experiment 2, we remove the pictures and instead prime mechanistic thinking with true-or-false questions about mechanism or function unrelated to the device itself. To foreshadow our results, we find that both manipulations produce the same effect: metacognitive judgments of understanding are lower when mechanistic knowledge is primed.

## Experiment 1

### Methods

#### Participants

Three hundred twenty-nine undergraduate students completed the experiment for course credit. Participants were aged 18–39 (*M* = 18.8 years). There were 185 female, 142 male, and 2 non-binary participants. Thirty-eight participants identified as Spanish, Hispanic, or Latino. Two hundred five participants identified as white, 51 as Asian, 31 as Black, 6 as American Indian or Alaskan Native, 1 as Native Hawaiian or Pacific Islander, 2 did not disclose, and 33 as other or more than one race. Participants were randomly assigned to one of two conditions: the *parts* condition (*n* = 164) or the *whole* condition (*n* = 165). Participants completed the experiment on a computer (remotely) at their own pace. The median duration of the experiment was 15.8 minutes. Data from all participants were used in analysis (no exclusion criteria were used).

#### Materials and Procedure

##### Understanding Task

First, participants rated their understanding of 41 man-made devices by answering the question “*How well do you understand how a ___ works?*” Devices included a *car, stapler, ballpoint pen, kite, air fryer, desk fan*, and more (see Figure 3 for a full list). Ratings were indicated on a seven-point Likert scale ranging from “*Not at all*” to “*Extremely well*” (midpoint “*Somewhat*”). Each device was displayed on its own page, along with a picture of the device. The pictures were obtained from Internet searches and are variable in both image quality and composition, wherein some are professional product photos and neatly organized, while others are photos in an everyday environment with no organization. The full stimuli and analysis code are available in the supplementary material.

**Figure 1:**
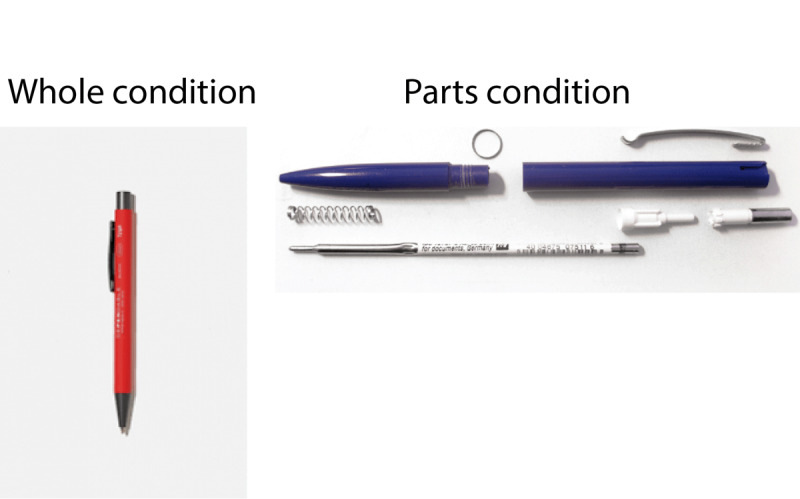
Participants in the whole condition (left) saw devices as whole objects in their typical form, while those in the parts condition (right) saw devices with their parts exposed.

Participants in the parts condition saw a picture of the device with its parts exposed. For example, a car with its hood propped open exposing the engine, a stapler that has been disassembled into six pieces, or a ballpoint pen taken apart (see Figure 1 for an example). Participants in the whole condition saw a picture of the device as a single object whose internal parts were not visible. The parts condition was intended to make the internal mechanisms of the device salient relative to the whole condition. The order in which devices were presented was fully randomized for each participant.

##### Cognitive Reflection Test

After the understanding task, participants performed a modified version of the cognitive reflection test to measure analytical cognitive style (Baron et al., 2015). The test includes three questions that each have both an intuitive answer and a correct answer that requires one to override the initial intuitive response. For example, one question reads “*Soup and crackers cost $4.50 in total. The soup costs $4 more than the crackers. How much do the crackers cost?*” (intuitive answer: $0.50, correct answer: $0.25). All three questions were presented on the same page. The task is scored as the total number of correct responses for each participant (range 0–3).

We include this measure as a covariate because previous studies have found that cognitive reflection is associated with subjective understanding (Fernbach, Sloman, et al., 2013; Gaviria & Corrdor, 2021). For example, Rhodes et al. (2014) found that individuals who are low in cognitive reflection report a higher degree of mechanistic understanding after reading a flawed scientific report compared to those high in cognitive reflection. This finding suggests that those who are low in cognitive reflection might be less scrutinous of their own understanding, perhaps driven by a tendency to overlook whether a factor is pertinent to an unbiased judgment of understanding.

##### Backwards Digit Span

Participants then completed the backwards digit span task. This task is a measure of working memory. Participants watch a sequence of digits on the screen, one at a time, at a rate of one second per digit. After the last digit appears, participants were asked to recall the sequence backwards. Digit sequences ranged from three to nine digits, with each sequence length being performed twice for a total of fourteen sequences (Berman et al., 2008). Random digit sequences were pre-generated, with the constraint that two identical digits could not appear sequentially. The task was scored as the total number of sequences recalled backwards correctly in their entirety (range 0–14).

We included this measure because low working memory is associated with higher susceptibility to seductive details (Sanchez & Wiley, 2006). In our experiment, the images of devices are irrelevant to one’s true understanding, but may lead those with low working memory capacity to have an inflated sense of understanding.

##### Need for Cognition

Lastly, participants completed a need for cognition assessment, which measures the degree to which an individual likes to engage in effortful cognition (Cacioppo & Petty, 1982; Cacioppo et al., 1984). The scale consists of 18 statements, such as “*I prefer simple to complex problems*”, and participants decide to what extent the statements are characteristic or uncharacteristic of themselves on a five-point Likert scale.

Need for cognition has been employed in related work and found to moderate ratings of subjective understanding. For example, Fernbach, Sloman, et al. (2013) found that those high in need for cognition derived a greater sense of understanding from explanations as they increased in detail, while those low in need for cognition did not. Analogously, the detailed images in the parts condition might cause those who are high in need for cognition to reflect more on their understanding compared to those who are low in need for cognition. At the end of the experiment, participants completed a demographic questionnaire.

### Results

**Figure 2:**
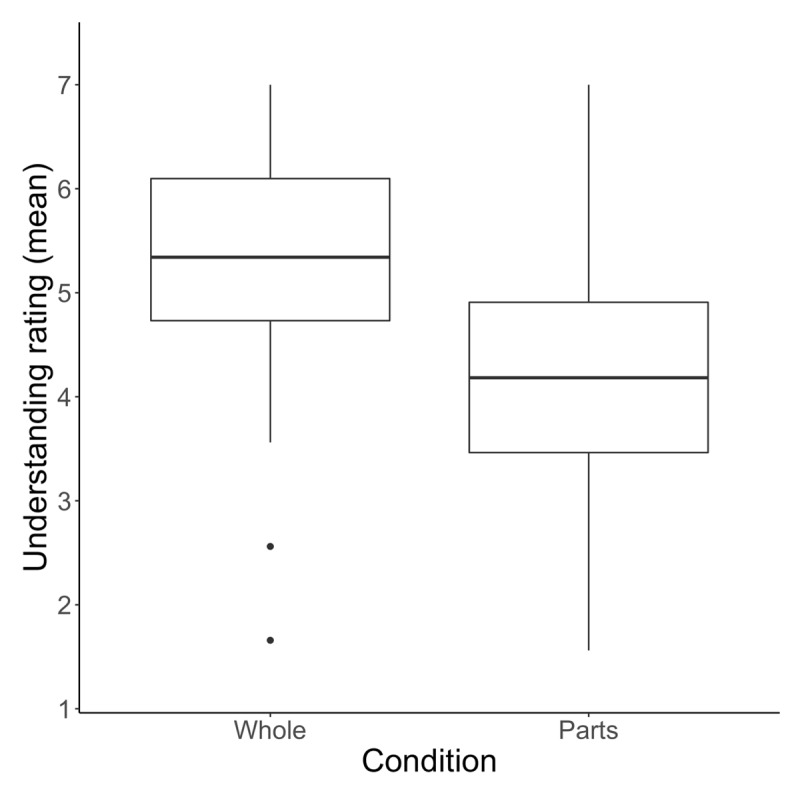
Participants in the whole condition rated their understanding of devices as higher than those in the parts condition.

**Figure 3:**
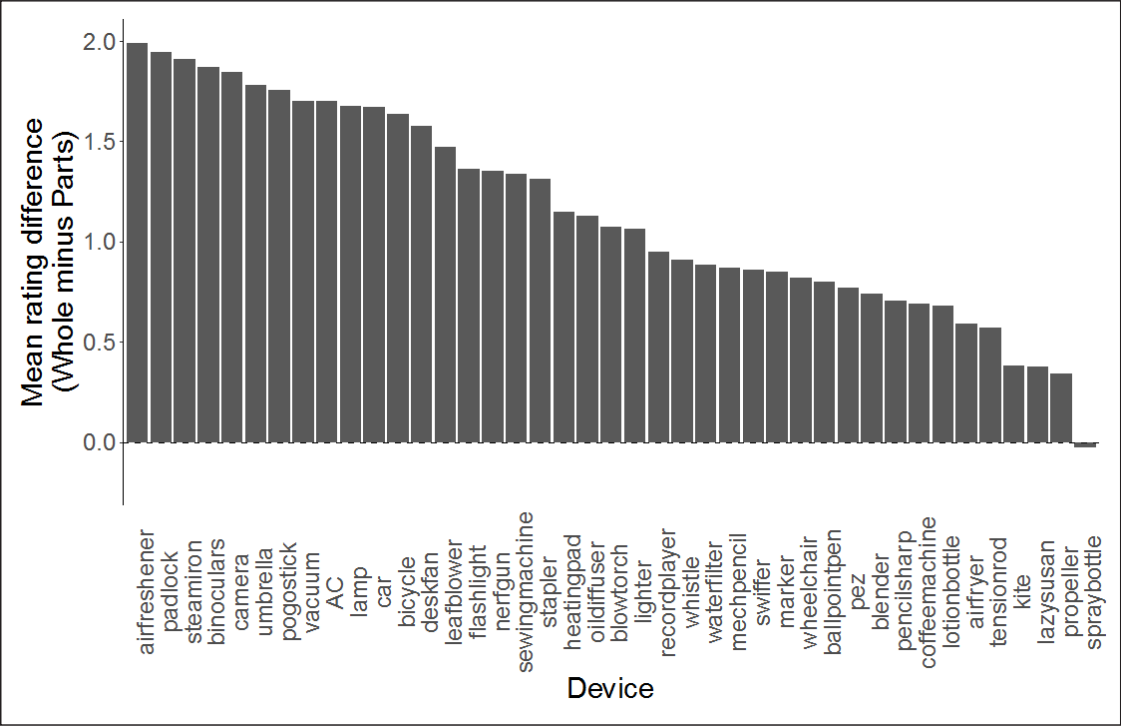
Participants rated their understanding as higher in the whole condition for every device except the spray bottle, which showed virtually no difference between conditions. Devices are sorted by mean difference between the two conditions (largest to smallest). An alternative visualization plotting the raw scores for both conditions is available in the supplementary material.

Participants in the whole condition rated their understanding of devices as greater than those in the parts condition, *M*_whole_ = 5.37 (*SD* = 1.71), *M*_parts_ = 4.22 *(SD* = 1.91). See Figure 2. Though understanding ratings varied by device, 40 of 41 devices were rated as higher (on average) in the whole condition (*p* < .001 by binomial test). The lone exception was the spray bottle, which showed almost no difference between conditions. See Figure 3. A two-way mixed ANOVA was performed with task condition (parts or whole) as a between-subjects factor and device (e.g., car, stapler, kite) as a within-subjects factor. The analysis revealed an interaction between device and task condition on understanding ratings, *F*(40, 13080) = 12.6, *p* < .001, \[\eta _G^2\] = .024, as well as a main effect of device, *F*(40, 13080) = 95.6, *p* < .001, \[\eta _G^2\] = .158 and a main effect of task condition, *F*(1, 327) = 111.7, *p* < .001, \[\eta _G^2\] = .109.

We conducted three separate regression analyses to examine whether the backwards digit span task, cognitive reflection test, and need for cognition scale were associated with the understanding ratings of each participant. Task condition was treated as a second predictor in each model, and device and participant were treated as random intercepts. Lower ratings of understanding were associated with higher scores on the cognitive reflection test, *t*(326.0) = –2.59, *p* = .01, and higher scores on the backwards digit span task, *t*(326.0) = –2.19, *p* = .029, but were not associated with scores on the need for cognition scale (*p* = .36). A separate regression analysis was performed using task condition and all three secondary tasks as predictors in a single regression model. In this model, scores on the cognitive reflection test were still associated with understanding ratings, *t*(324.0) = –2.02, *p* = .045, but backwards digit span and need for cognition were not (both *p*s > .10). Task condition remained a significant predictor in all four models (all *p* < .001).

### Discussion

In Experiment 1, participants rated their understanding of 41 man-made devices. Though participants in both conditions were asked the same question, those who saw pictures of the internal parts of the device rated their understanding as less than those who saw an external, whole-object view of the device. The effect was consistent in direction for nearly all of the devices rated. This result revealed that making the internal mechanisms salient through pictorial cues led to lower judgments of understanding.

Individual differences in cognition were also associated with judgments of understanding. Those with lower working memory capacity and those who engaged in an intuitive cognitive reasoning style rated their understanding of the devices as higher than those with greater working memory and those who used an analytical reasoning style, respectively. These results are consistent with predictions derived from prior literature (Fernbach, Sloman, et al., 2013; Gaviria & Corrdor, 2021; Rhodes, et al., 2014; Sanchez & Wiley, 2006). Notably, a self-reported tendency to engage in effortful thought (need for cognition) was not associated with understanding ratings.

## Experiment 2

In Experiment 1, participants who saw the internal parts of a device rated their understanding lower than those who saw an external whole-object view of the device. To remind the reader, our hypothesis was that the parts condition led participants to evaluate their mechanistic understanding of the device, whereas participants in the whole condition also evaluated their functional understanding of the device (i.e., how to operate the device and what it is used for). An alternative hypothesis is that participants in both conditions adopted a mechanistic mindset, but that those in the parts condition rated their understanding as lower after being confronted with evidence that their understanding was incomplete (e.g., they saw unfamiliar parts or assembly). That is, the pictures in the parts condition contained information about how the devices worked, and this may have dispelled a sense of understanding. The quality of the pictures themselves may have also influenced ratings. Because the pictures were obtained from Internet searches, they were not carefully matched for context or organization. For example, some devices were shown on a blank white background while others were shown on a desk or other natural settings. In addition, some of the images in the parts condition showed each part neatly laid out and organized, while others showed parts laid out haphazardly or as part of the device itself (e.g., the inside of a sewing machine, but not disassembled).

In Experiment 2, we addressed these concerns with a different manipulation. Participants answered true-or-false questions about mechanistic or functional statements that were unrelated to the devices themselves in order to induce a mechanistic or functional mindset. A third control condition contained no true-or-false questions to measure participants’ subjective understanding in a default framing. In this way, no information about how the devices worked was revealed to participants in any of the conditions.

### Methods

#### Participants

Three hundred eighty-one undergraduate students completed the experiment for course credit. Participants were aged 18–37 (*M* = 18.7 years, 12 unknown age). There were 209 female, 166 male, and 6 non-binary participants. Thirty-eight participants identified as Spanish, Hispanic, or Latino. Two hundred sixty-seven participants identified as white, 79 as Asian, 30 as Black, 5 as American Indian or Alaskan Native, 2 as Native Hawaiian or Pacific Islander, 8 did not disclose, and 36 as other or more than one race. Data from all participants were used in analysis (no exclusion criteria were used).

#### Design and Procedure

Participants were randomly assigned to one of three conditions: mechanistic (*n* = 121), functional (*n* = 125), or control (*n* = 135). Participants rated their understanding of the same 41 devices^2^ as in Experiment 1, one device per page, in a random order. Each device was paired with a true-or-false statement that either probed functional or mechanistic knowledge depending on the participant’s condition. For example, one functional statement read: “*People use alarm clocks in order to wake up on time*.” One mechanistic statement read: “*Scars heal because the body releases serotonin into the wound*.” The statements were constructed so that half were true and half were false. No feedback to the true-or-false questions was provided, and no pictures of the devices were shown. None of the statements referenced any of the 41 devices for which participants rated their understanding. That is, these statements were intended to promote a mechanistic or functional mindset, but were otherwise unrelated to the primary task. In the control condition, participants only rated their understanding of the devices and did not see any true-or-false statements. The full stimuli and analysis code are available in the supplementary material.

After rating their understanding of each device, participants were tested on their memory for devices that were encountered in the experiment with four multiple-choice questions. For each question, participants were asked to select the device they recalled seeing during the experiment (among four options). Each participant was given a score (range 0-4) indicating how many questions they answered correctly. Then, participants completed the cognitive reflection test, backwards digit span test, and need for cognition scale, which all followed the same presentation order as in Experiment 1. Finally, participants completed a demographic questionnaire. Participants completed the experiment on a computer (remotely) at their own pace. The median duration of the experiment was 18.8 minutes.

### Results

A two-way mixed ANOVA was performed with task condition (mechanistic vs. functional vs. control) as a between-subjects factor and device (e.g., car, stapler, kite) as a within-subjects factor. The analysis revealed an interaction between device and task condition on understanding ratings, *F*(80, 15120) = 1.28, *p* = .048, \[\eta _G^2\] = .005, though this did not survive a Greenhouse-Geisser sphericity correction (*p* = .09). The analysis also revealed a main effect of device, *F*(40, 15120) = 93.2, *p* < .001, \[\eta _G^2\] = .143, and a main effect of task condition, *F*(2, 378) = 3.36, *p* = .036, \[\eta _G^2\] = .006.

**Figure 4:**
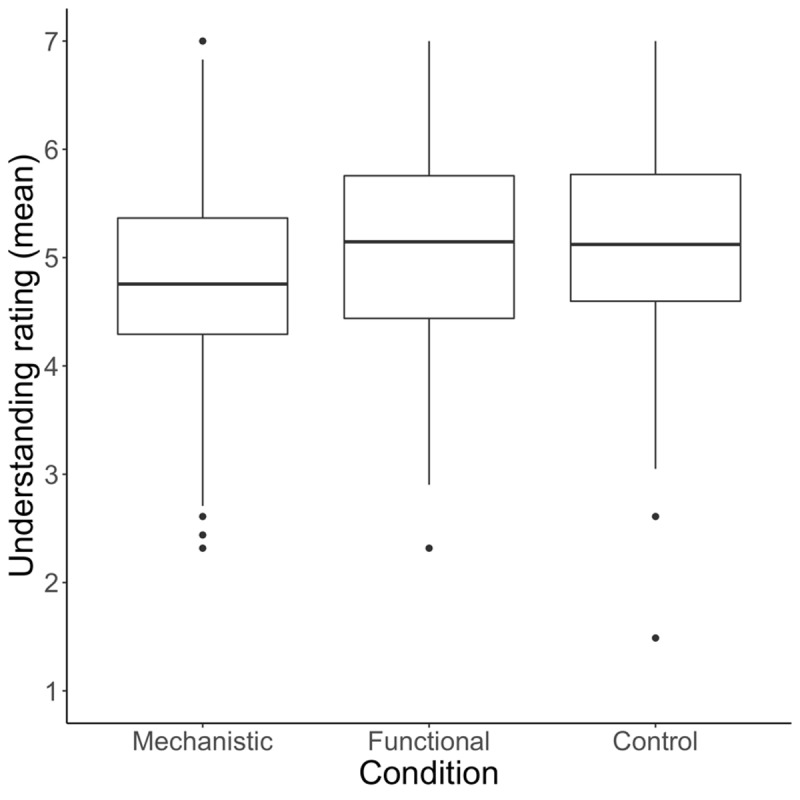
Participants in the mechanistic condition rated their understanding of devices as lower than those in the functional and control conditions.

**Figure 5:**
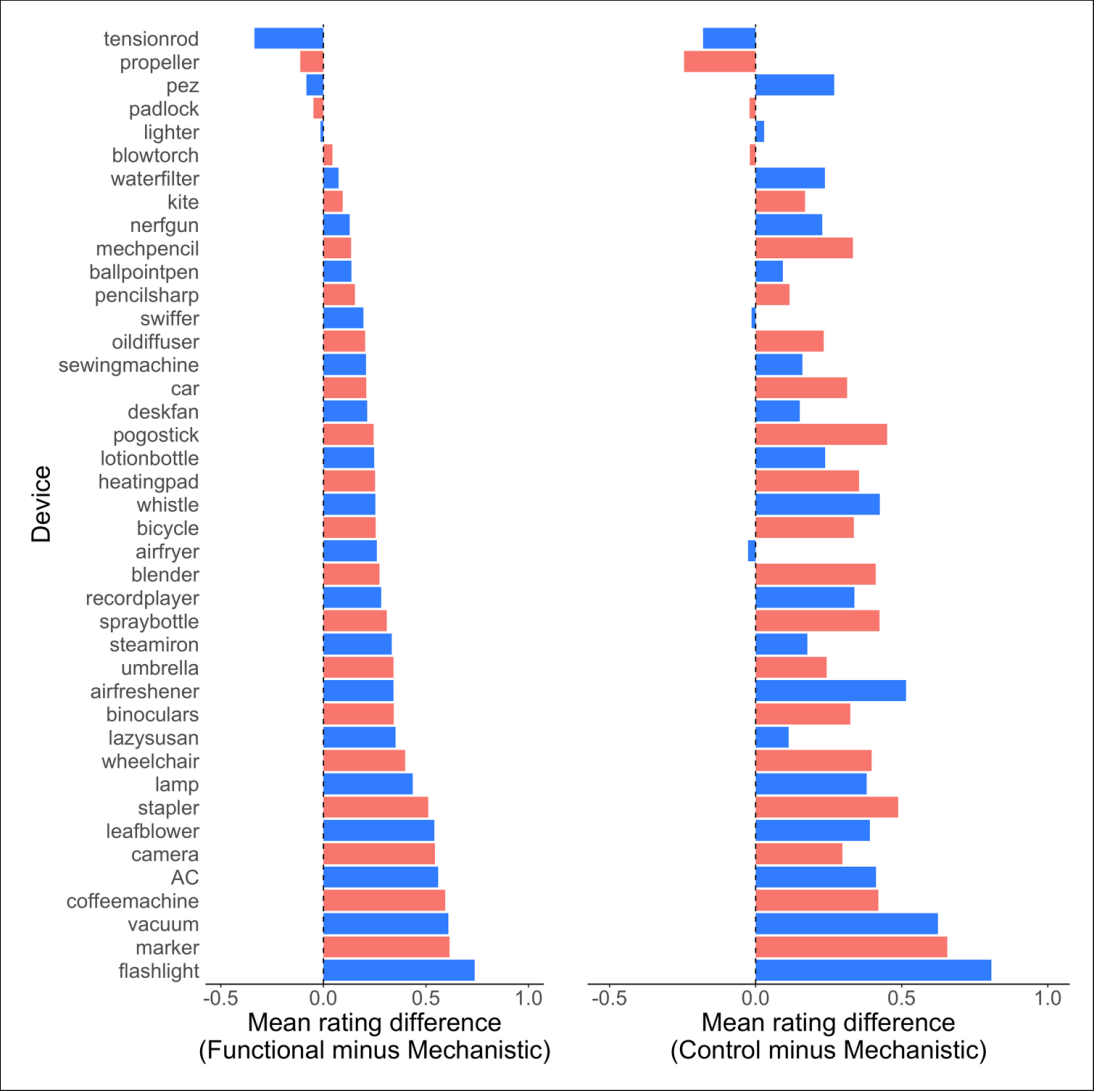
Participants rated their understanding as lowest in the mechanistic condition for 35 of 41 devices. Devices are sorted by mean difference between the functional and mechanistic conditions (smallest to largest). Bar colors alternate solely to aid in visual comparison between conditions. An alternative visualization plotting the raw scores in each condition is available in the supplementary material.

The main effect of condition appeared to be driven by lower ratings in the mechanistic condition (*M*_mechanistic_ = 4.84, *SD* = 1.79) relative to the control (*M*_control_ = 5.11, *SD* = 1.79) and functional (*M*_functional_ = 5.10, *SD* = 1.75) conditions. See Figure 4. This idea was confirmed by additional ANOVAs that found a pairwise difference in ratings between the functional and mechanistic conditions, *F*(1, 244) = 4.81, *p* = 0.03, and between mechanistic and control conditions, *F*(1, 254) = 5.27, *p* = .023, but not between the control and functional conditions (*p* = .96). Mean ratings were lower in the mechanistic than functional condition for 36 of 41 devices (*p* < .001 by binomial test), and lower in the mechanistic than the control condition for 35 of 41 devices (*p* < .001 by binomial test). The exceptions were: tension rod, propeller, pez dispenser, padlock, and lighter (rated higher in the functional than mechanistic condition); tension rod, propeller, padlock, blow torch, swiffer, and air fryer (rated higher in the control than mechanistic condition). See Figure 5.

The true-or-false manipulation was intended to affect how participants evaluate their understanding of the devices themselves. An alternative hypothesis is that the effect is not device specific: participants may be overconfident in general, and the mechanistic condition may lead to calibration of this overconfidence because the mechanistic true-or-false statements are more difficult than the functional ones. However, the differences in understanding ratings of Experiment 1 (parts minus whole) were correlated with the differences in understanding ratings of Experiment 2 (mechanistic minus functional), *r*(39) = .32, *p* = .04, providing some evidence that the manipulations in both Experiment 1 and 2 caused participants to re-evaluate their understanding of the devices themselves, rather than a general reduction in overconfidence. One limitation of this analysis is that it is cross-experimental, involving different participant samples.

We conducted three separate regression analyses to see whether individual differences in cognitive reflection, need for cognition, and backwards digit span were associated with the understanding ratings of each participant. Task condition was treated as a second predictor in each model, and device and participant were treated as random intercepts. Lower ratings of understanding were associated with *lower* scores on the need for cognition scale, *t*(377.0) = 2.00, *p* = .047, but were not associated with backwards digit span or cognitive reflection (both *p*s > .39).

On the memory test, 85% of participants correctly answered all four questions correctly (*M* = 3.69), indicating that most participants engaged with the task. Participants who had better memory for the devices also reported higher levels of understanding, after controlling for task condition, device, and participant, *t*(377.0) = 3.52, *p* < .001.

### Discussion

In Experiment 2, we found additional support for our hypothesis that emphasizing mechanistic knowledge affects self-reported judgements of understanding. Participants who answered true-or-false questions about mechanistic knowledge rated their understanding of devices as lower than participants who were tested on functional knowledge. We found no difference between the control and functional conditions, which may indicate that people use functional understanding as a default framing for understanding judgments, though we caution drawing strong inferences from a null effect.

Unlike Experiment 1, individual differences in cognition measured using the cognitive reflection test and backwards digit span were not associated with understanding ratings. However, those who self-reported a higher need for cognition did indicate greater self-reported judgments of understanding. This finding was unexpected, as we believed that those high in need for cognition would be more critical of their own understanding and report lower judgments.

## General Discussion

In two experiments, we found evidence that participants rated their understanding of man-made devices lower when mechanistic knowledge was primed. In Experiment 1, a mechanistic mindset was induced through pictures that revealed the internal parts of a device. In Experiment 2, a mechanistic mindset was induced using true-or-false statements that probed mechanistic knowledge. These results build on previous work demonstrating that self-assessments of understanding are malleable (Alter et al., 2010; Fisher et al., 2015; Fisher & Oppenheimer, 2021; Rozenblit & Keil, 2002; Sloman & Rabb, 2016; Walters et al., 2017). Our results suggest that people may fail to consider their knowledge of mechanisms when evaluating their own understanding of devices, instead relying on more easily accessible cues such as knowledge of a device’s function. Although previous results have hinted at this possibility (Alter et al., 2010; Fernbach, Rogers, et al., 2013; Kelemen, Rottman, & Seston, 2013; Rozenblit & Keil, 2002), our experiments demonstrate the effect directly and outside of the illusion of explanatory depth paradigm. In addition, we introduce two novel manipulations to induce a mechanistic or functional mindset.

We also found that individual differences in cognition may influence subjective understanding, though the results were not consistent across experiments. Low working memory capacity and intuitive cognitive style were associated with higher judgments of understanding in Experiment 1, but not in Experiment 2. One explanation for this discrepancy is the nature of our manipulations. In Experiment 1, images presented to participants were akin to seductive details: while superficially relevant, it was not necessary to attend to them in order to provide judgments of understanding. These seductive details may have inflated subjective understanding in those who adopted an intuitive cognitive style and those low working memory capacity, despite objective understanding being positively correlated with working memory capacity (Just & Carpenter, 1992). Consistent with this possibility, Sanchez and Wiley (2006) found that those with low working memory capacity attend to seductive images more often than those with high working memory capacity.

In contrast to the first experiment, Experiment 2 required participants to judge whether statements were true or false. Participants were required to engage with these stimuli, but the statements themselves did not relate to the device ratings, as the statements and ratings were presented separately. Because there was no apparent logical connection between the statements and devices, they may not have acted as seductive details that might influence subjective understanding. Conversely, we found that higher need for cognition was associated with higher understanding ratings for Experiment 2 but not in Experiment 1. This association was in the opposite direction of what was expected.

It is somewhat surprising that these individual difference measures were not consistently associated with subjective understanding across the two experiments, especially because task condition was treated as a separate controlling factor. In previous work, each of these individual differences (working memory, need for cognition, and cognitive reflection) have been associated with subjective understanding, although using different experimental designs (Fernbach, Sloman, et al., 2013; Gaviria & Corrdor, 2021; Rhodes, et al., 2014; Sanchez & Wiley, 2006). On the other hand, the results from our two experiments do not contradict each other: for each measure, a significant result in one measure for one experiment is paired with a failure to reject the null for that measure in another experiment. While these measures were not the primary focus of the present work, future studies could explore the boundary conditions in which they are associated with judgments of understanding.

### Theory and Limitations

Our theory is that people hold multiple levels of understanding a complex system, including a functional understanding and a mechanistic one. While people generally have a good sense of what artifacts do, they often have a poor understanding of how they work (Rozenblit & Keil, 2002). When a person is asked to rate their understanding of an artifact they may adopt either a functional or mechanistic mindset depending on which cues are most accessible. We argue that making mechanistic information more salient leads to adoption of a mechanistic mindset, resulting in lower ratings of understanding. However, our experimental designs leave open some alternative possibilities.

In Experiment 1, we primed participants to think about function or mechanism by presenting pictures of devices in a whole-object view or decomposed into parts. We argue that the pictures of a device’s parts increased the salience of mechanism, which led to lower ratings of understanding. However, it is possible that participants reported lower ratings because they encountered parts or assembly that were unfamiliar to them, wherein participants might have rationally revised their beliefs when confronted with evidence that they did not understand how the device worked. It is also possible that the manipulation worked because it focused participants’ attention on parts, but not mechanism per se. For example, participants who saw a picture of a ballpoint pen’s parts might have considered whether they understand the *function* of each part—though it is difficult to explain the function of a part without appeal to the mechanism of the whole (McCarthy & Keil, 2023).

In Experiment 2, we primed participants to think about function or mechanism by asking them to answer true-or-false questions related to mechanistic or functional knowledge. Our hypothesis is that these questions induced a mechanistic or functional mindset that subsequently affected how participants rated their own understanding. An alternative hypothesis is that since the functional questions tended to be less difficult than the mechanistic ones, participants rated their understanding lower in the mechanistic condition because their competence is challenged. Critically, under this explanation, the lower rating does not have anything to do with mechanistic knowledge per se. At the same time, it is not very surprising that people were more accurate for functional true-or-false questions because people actually *do* understand function better than mechanism. If people understood function and mechanism equally well, we would expect no difference in understanding ratings regardless of mindset. As such, it is difficult to equate the difficulty of function and mechanism true-or-false questions. A future experiment could test the alternative hypothesis with a different manipulation. For example, participants could solve difficult math problems that challenge their competence and see if it causes a similar drop in understanding ratings. This hypothetical experiment could provide support for the alternative hypothesis that challenging one’s competence leads to lower ratings of understanding, but the two hypotheses are not mutually exclusive.

Another prediction from our theory is that in cases where a person has a good understanding of an artifact’s mechanism but does not understand its function, they may actually provide a *higher* rating of understanding when primed with mechanistic detail. However, it may be difficult for participants to neglect function entirely because artifacts are designed to fulfill some function, and understanding of the mechanism implies that function. Cases where a device’s mechanism is understood better than its function are rare in the world, and exceptions are noteworthy. Consider the Antikythera mechanism, an ancient (and anachronistic) Greek mechanical device. The device has fascinated archaeologists in part because its precise function is unknown (Edmunds & Morgan, 2000). As modern imaging techniques reveal more of the device’s parts and inscriptions, theories of its function have become more precise—from an “astronomical or calendrical calculating device” (de Solla Price, 1974) to a realization of Hipparchos’ lunar theory (Freeth et al., 2006).

Our results also leave open questions regarding the boundaries and implications of the effect. Our experimental stimuli include only man-made devices, where knowledge of function and mechanism may be conflated (e.g., one may understand how to operate a TV remote control, but not understand how the internal parts of a TV remote control enable it to operate). In contrast, adults do not view non-biological natural kinds as having a purpose (Kelemen 1999a). For example, one could provide a mechanistic explanation for “how a volcano works,” but it is difficult to imagine what a functional explanation would look like. If a failure to distinguish between these two types of knowledge is partially responsible for the observed effect, our findings should not generalize to non-biological natural kinds that do not have prescribed functions in the same way as artifacts. However natural kinds like biological parts and evolutionary traits can serve a function and are sometimes viewed as having a purpose despite not being designed for one (Kelemen, 2012; McCarthy & Keil, 2023). It seems natural to explain how a heart works by appealing to its function of circulating blood, even if that function was never prescribed. If our results generalize to these domains, it may reinforce a stance that people view biological parts and evolutionary traits as existing to serve some function. For example, one hypothesis is that the effect does generalize to biological natural kinds, but is more pronounced in those who endorse teleological explanations of such kinds than those who do not (Kelemen & Rosset, 2009).

We have also not tested whether mechanistic priming has any downstream effects on cognition. For example, if mechanistic priming leads participants to recognize their lack of understanding, it may lead to increased information seeking behaviors or have implications for studying in educational settings.

Our understanding of artifacts is multifaceted and includes both functional and mechanistic understanding. How we judge our own understanding can impact how we view the world. Our findings cast light on the nature of those judgments, and how they are affected by context that promotes a mechanistic or functional mindset. While an unbiased evaluation of one’s own understanding is complex, mechanistic knowledge is surely an important element that is underweighted in this process.

## Data Accessibility Statement

The raw data, analysis code, experimental stimuli, and additional figures (where noted in the manuscript) are available on OSF: https://osf.io/a8rjv.

## Footnotes

The study contained only natural phenomena as stimuli. To conduct this analysis, Rozenblit and Keil (2002) treated data from prior experiments in the paper as a separate condition.

The labels of some devices were changed to minimize ambiguity because, unlike Experiment 1, no images were presented. For example, “vent clip air freshener” was changed to “car vent clip air freshener.”

## References

[1] Alter, A. L., & Oppenheimer, D. M. (2009). Uniting the tribes of fluency to form a metacognitive nation. Personality and Social Psychology Review, 13(3), 219–235. DOI: 10.1177/1088868309341564 19638628

[2] Alter, A. L., Oppenheimer, D. M., & Zemla, J. C. (2010). Missing the trees for the forest: a construal level account of the illusion of explanatory depth. Journal of Personality and Social Psychology, 99(3), 436–451. DOI: 10.1037/a0020218 20658836

[3] Baron, J., Scott, S., Fincher, K., & Metz, S. E. (2015). Why does the Cognitive Reflection Test (sometimes) predict utilitarian moral judgment (and other things)? Journal of Applied Research in Memory and Cognition, 4(3), 265–284. DOI: 10.1016/j.jarmac.2014.09.003

[4] Berman, M. G., Jonides, J., & Kaplan, S. (2008). The cognitive benefits of interacting with nature. Psychological Science, 19(12), 1207–1212. DOI: 10.1111/j.1467-9280.2008.02225.x 19121124

[5] Cacioppo, J. T., & Petty, R. E. (1982). The need for cognition. Journal of Personality and Social Psychology, 42(1), 116–131. DOI: 10.1037/0022-3514.42.1.116

[6] Cacioppo, J. T., Petty, R. E., & Feng Kao, C. (1984). The efficient assessment of need for cognition. Journal of Personality Assessment, 48(3), 306–307. DOI: 10.1207/s15327752jpa4803_13 16367530

[7] de Solla Price, D. (1974). Gears from the Greeks. The Antikythera mechanism: a calendar computer from ca. 80 BC. Transactions of the American Philosophical Society, 1–70. DOI: 10.2307/1006146

[8] Edmunds, M. G., & Morgan, P. (2000). The Antikythera Mechanism: still a mystery of Greek astronomy? Astronomy & geophysics, 41(6), 6–10. DOI: 10.1046/j.1468-4004.2000.41610.x

[9] Fernbach, P. M., Rogers, T., Fox, C. R., & Sloman, S. A. (2013). Political extremism is supported by an illusion of understanding. Psychological Science, 24(6), 939–946. DOI: 10.1177/0956797612464058 23620547

[10] Fernbach, P. M., Sloman, S. A., Louis, R. S., & Shube, J. N. (2013). Explanation fiends and foes: How mechanistic detail determines understanding and preference. Journal of Consumer Research, 39(5), 1115–1131. DOI: 10.1086/667782

[11] Fisher, M., Goddu, M. K., & Keil, F. C. (2015). Searching for explanations: How the Internet inflates estimates of internal knowledge. Journal of Experimental Psychology: General, 144(3), 674–688. DOI: 10.1037/xge0000070 25822461

[12] Fisher, M., & Oppenheimer, D. M. (2021). Who knows what? Knowledge misattribution in the division of cognitive labor. Journal of Experimental Psychology: Applied, 27(2), 292–306. DOI: 10.1037/xap0000310 33829826

[13] Freeth, T., Bitsakis, Y., Moussas, X., Seiradakis, J. H., Tselikas, A., Mangou, H., … & Edmunds, M. G. (2006). Decoding the ancient Greek astronomical calculator known as the Antikythera Mechanism. Nature, 444(7119), 587–591. DOI: 10.1038/nature05357 17136087

[14] Gaviria, C., & Corredor, J. (2021). Illusion of explanatory depth and social desirability of historical knowledge. Metacognition and Learning, 16(3), 801–832. DOI: 10.1007/s11409-021-09267-7

[15] Garner, R., Gillingham, M. G., & White, C. S. (1989). Effects of ‘seductive details’ on macroprocessing and microprocessing in adults and children. Cognition and Instruction, 6(1), 41–57. DOI: 10.1207/s1532690xci0601_2

[16] Gopnik, A. (2000). Explanation as orgasm and the drive for causal knowledge: The function, evolution, and phenomenology of the theory formation system. In F. C. Keil & R. A. Wilson (Eds.), Explanation and cognition (pp. 299–323). The MIT Press. DOI: 10.7551/mitpress/2930.003.0018

[17] Harp, S. F., & Maslich, A. A. (2005). The consequences of including seductive details during lecture. Teaching of Psychology, 32(2), 100–103. DOI: 10.1207/s15328023top3202_4

[18] Harp, S. F., & Mayer, R. E. (1997). The role of interest in learning from scientific text and illustrations: On the distinction between emotional interest and cognitive interest. Journal of Educational Psychology, 89(1), 92–102. DOI: 10.1037/0022-0663.89.1.92

[19] Haskel-Ittah, M. (2023). Explanatory black boxes and mechanistic reasoning. Journal of research in science teaching, 60(4), 915–933. DOI: 10.1002/tea.21817

[20] Ikeda, K., Kitagami, S., Takahashi, T., Hattori, Y., & Ito, Y. (2013). Neuroscientific information bias in metacomprehension: The effect of brain images on metacomprehension judgment of neuroscience research. Psychonomic Bulletin & Review, 20, 1357–1363. DOI: 10.3758/s13423-013-0457-5 23728726

[21] Joo, S., Yousif, S. R., & Keil, F. C. (2022). Understanding “why:” how implicit questions shape explanation preferences. Cognitive Science, 46(2), e13091. DOI: 10.1111/cogs.13091 35122293

[22] Jordan, K., Zajac, R., Bernstein, D., Joshi, C., & Garry, M. (2022). Trivially informative semantic context inflates people’s confidence they can perform a highly complex skill. Royal Society Open Science, 9(3), 211977. DOI: 10.1098/rsos.211977 35308623 PMC8924756

[23] Just, M. A., & Carpenter, P. A. (1992). A capacity theory of comprehension: individual differences in working memory. Psychological Review, 99(1), 122–149. DOI: 10.1037/0033-295X.99.1.122 1546114

[24] Kahneman, D., & Frederick, S. (2002). Representativeness revisited: Attribute substitution in intuitive judgment. Heuristics and biases: The psychology of intuitive judgment, 49–81. DOI: 10.1017/CBO9780511808098.004

[25] Keil, F. (2006). Explanation and understanding. Annual Review of Psychology, 57, 227–254. DOI: 10.1146/annurev.psych.57.102904.190100 PMC3034737 16318595

[26] Keil, F. (2019). How do partial understandings work. Varieties of understanding: New perspectives from philosophy, psychology, and theology, 191–208. DOI: 10.1093/oso/9780190860974.003.0010

[27] Kelemen, D. (1999a). Function, goals and intention: Children’s teleological reasoning about objects. Trends in Cognitive Sciences, 3(12), 461–468. DOI: 10.1016/S1364-6613(99)01402-3 10562725

[28] Kelemen, D. (1999b). Why are rocks pointy? Children’s preference for teleological explanations of the natural world. Developmental Psychology, 35(6), 1440–1452. DOI: 10.1037//0012-1649.35.6.1440 10563733

[29] Kelemen, D. (2012). Teleological minds: How natural intuitions about agency and purpose influence learning about evolution. In K. S. Rosengren, S. K. Brem, E. M. Evans, & G. M. Sinatra (Eds.), Evolution challenges: Integrating research and practice in teaching and learning about evolution (pp. 66–92). Oxford: Oxford University Press. DOI: 10.1093/acprof:oso/9780199730421.003.0004

[30] Kelemen, D., & Rosset, E. (2009). The human function compunction: Teleological explanation in adults. Cognition, 111(1), 138–143. DOI: 10.1016/j.cognition.2009.01.001 19200537

[31] Kelemen, D., Rottman, J., & Seston, R. (2013). Professional physical scientists display tenacious teleological tendencies: purpose-based reasoning as a cognitive default. Journal of Experimental Psychology: General, 142(4), 1074–1083. DOI: 10.1037/a0030399 23067062

[32] Kelley, C. M., & Lindsay, D. S. (1993). Remembering mistaken for knowing: Ease of retrieval as a basis for confidence in answers to general knowledge questions. Journal of Memory and Language, 32(1), 1–24. DOI: 10.1006/jmla.1993.1001

[33] Koriat, A. (1997). Monitoring one’s own knowledge during study: A cue-utilization approach to judgments of learning. Journal of Experimental Psychology: General, 126(4), 349–370. DOI: 10.1037/0096-3445.126.4.349

[34] Lawson, R. (2006). The science of cycology: Failures to understand how everyday objects work. Memory & Cognition, 34(8), 1667–1675. DOI: 10.3758/BF03195929 17489293

[35] Lombrozo, T., & Wilkenfeld, D. (2019). Mechanistic versus functional understanding. Varieties of Understanding: New Perspectives from Philosophy, Psychology, and Theology, 209–229. DOI: 10.1093/oso/9780190860974.003.0011

[36] McCarthy, A. M., & Keil, F. C. (2023). A right way to explain? function, mechanism, and the order of explanations. Cognition, 238, 105494. DOI: 10.1016/j.cognition.2023.105494 37270890

[37] Nickerson, R. S., & Adams, J. J. (1979). Long-term memory for a common object. Cognitive Psychology, 11, 287–307. DOI: 10.1016/0010-0285(79)90013-6

[38] Rozenblit, L., & Keil, F. (2002). The misunderstood limits of folk science: An illusion of explanatory depth. Cognitive Science, 26(5), 521–562. DOI: 10.1207/s15516709cog2605_1 21442007 PMC3062901

[39] Rhodes, R. E., Rodriguez, F., & Shah, P. (2014). Explaining the alluring influence of neuroscience information on scientific reasoning. Journal of Experimental Psychology: Learning, Memory, and Cognition, 40(5), 1432–1440. DOI: 10.1037/a0036844 24820673

[40] Sanchez, C. A., & Wiley, J. (2006). An examination of the seductive details effect in terms of working memory capacity. Memory & Cognition, 34, 344–355. DOI: 10.3758/BF03193412 16752598

[41] Sloman, S. A., & Rabb, N. (2016). Your understanding is my understanding: Evidence for a community of knowledge. Psychological Science, 27(11), 1451–1460. DOI: 10.1177/0956797616662271 27670662

[42] Vasilyeva, N., Wilkenfeld, D., & Lombrozo, T. (2017). Contextual utility affects the perceived quality of explanations. Psychonomic Bulletin & Review, 24, 1436–1450. DOI: 10.3758/s13423-017-1275-y 28386858

[43] Walters, D. J., Fernbach, P. M., Fox, C. R., & Sloman, S. A. (2017). Known unknowns: A critical determinant of confidence and calibration. Management Science, 63(12), 4298–4307. DOI: 10.1287/mnsc.2016.2580

[44] Weisberg, D. S., Keil, F. C., Goodstein, J., Rawson, E., & Gray, J. R. (2008). The seductive allure of neuroscience explanations. Journal of Cognitive Neuroscience, 20(3), 470–477. DOI: 10.1162/jocn.2008.20040 18004955 PMC2778755

[45] Zemla, J. C., Steiner, S. M., & Sloman, S. (2016). Analytical thinking predicts less teleological reasoning and religious belief. In Proceedings of the 38th Annual Conference of the Cognitive Science Society (pp. 1217–1222). Austin, TX: Cognitive Science Society.

